# Cost-utility analysis of lenvatinib and sorafenib for the first-line treatment of unresectable hepatocellular carcinoma in Vietnam: Evidence from a lower-middle income country

**DOI:** 10.1371/journal.pone.0345212

**Published:** 2026-04-03

**Authors:** Thang Xuan Do, Ha Thi Nguyen, Phuong Thi Lan Nguyen, Mai Thi Tuyet Kieu, Tuan Viet Duong, Van Nu Hanh Pham

**Affiliations:** 1 Department of Pharmaceutical Management and Pharmacoeconomics, Hanoi University of Pharmacy, Hanoi, Vietnam; 2 University of Health Sciences, Vietnam National University Ho Chi Minh City, Ho Chi Minh, Vietnam; Xiangya Hospital Central South University, CHINA

## Abstract

**Objectives:**

To evaluate cost-effectiveness of lenvatinib compared with sorafenib as first-line treatment in unresectable hepatocellular carcinoma (HCC) patients in Vietnam from a perspective of the public third-party payer.

**Methods:**

A three-health state partitioned survival model was developed to compare costs and health outcomes of lenvatinib and sorafenib in unresectable advanced HCC patients over a 10-year time horizon, using a cycle length of 28-day. Clinical data on efficacy and safety of lenvatinib and sorafenib from the REFLECT trial was used to extrapolate outcomes beyond the follow-up period, while medication costs and health care resources were estimated based on local data and clinical experts’ consultations. A discount rate of 3% was applied to both costs and outcomes. Parameter uncertainty was explored using one-way sensitivity analysis and probabilistic sensitivity analysis. The impact of different time horizons and different approaches to extrapolate survival time were examined in scenario analyses.

**Results:**

In unresectable HCC patients, lenvatinib gained more 0.28 life-years (LYs) equaling to 0.21 quality-adjusted life-years (QALYs) compared with sorafenib. Without consideration of reimbursement rate for all medications and co-insurance, usage of lenvatinib led to an increase of 3,451.3 USD. Therefore, lenvatinib was not cost-effective compared with sorafenib, with an incremental cost-effectiveness ratio (ICER) of 16,114.5 USD/QALY. However, if lenvatinib was reimbursed at the same rate as sorafenib (50%), it became cost-effective, with an ICER of 8,307.6 USD/QALY. Results from the base-case analysis were robust in different sensitivity analyses.

**Conclusions:**

With a significant improvement in progression-free survival, lenvatinib gained more LYs and QALYs compared with sorafenib. At the same reimbursement rate of sorafenib of 50%, lenvatinib was cost-effective from the perspective of the third-party payer.

## Introduction

Liver cancer is the fifth most common cancer (866,136 cases, accounting for 4.3%) and the third leading cause of cancer-related death (758,725 cases, accounting for 7.8%) worldwide [1]. Notably, more than 90% of primary liver cancer were hepatocellular carcinoma (HCC) [2]. In Vietnam – a lower-middle income country (LMIC) with high prevalence of hepatitis B virus (HBV) infection, liver cancer was the second most common cancer and the leading cause of cancer-related death [3]. In 2022, there were 24,502 new cases and 23,333 patients died from liver cancer [3]. Recent studies have also emphasized evolving therapeutic strategies and molecular insights in HCC, reflecting the dynamic treatment landscape in which economic evidence is increasingly essential [4–6].

Notably, according to a 7-year survey in Vietnam, about 40.8% of HCC patients were diagnosed at an advanced stage, not amenable to resection or locoregional therapy [7]. Vietnamese Ministry of Health (MoH) issued an updated guideline for diagnosis and treatment of HCC patients in 2020, which recommended the use of systemic therapy (i.e., sorafenib and lenvatinib) as first-line treatment for unresectable advanced HCC with ECOG Performance Status (ECOG PS) 1–2 and Child Pugh A/B. The pivotal trial REFLECT [8] conducted in 1,492 unresectable HCC, indicated that compared with sorafenib, lenvatinib presented non-inferiority in terms of overall survival (OS) and significantly prolonged progression-free survival (PFS) with HR = 0.66 (95% confident interval (95%CI) 0.57–0.77). Evidence from real-world data in the Asian region [9–16] re-confirmed the benefit and safety profile of lenvatinib.

In the context of Vietnam, sorafenib was reimbursed in 2011. Since then, no systemic therapy in the first-line setting was included in the benefit package. Lenvatinib was authorized by MoH in 2020 but has not been reimbursed yet. Therefore, advanced HCC patients with the health insurance card in Vietnam could only access to sorafenib or had to pay out-of-pocket to use lenvatinib. No cost-effectiveness evidence existed to support for decision making on reimbursement of lenvatinib in Vietnam. Previous studies evaluating cost-effectiveness of lenvatinib over sorafenib were conducted primarily in high-income countries (HIC) [17–21] and in China – an upper-middle-income country (UMIC) [22,23]. It should be noted that the acquisition cost of lenvatinib was much lower than that of sorafenib in almost studies [17–21] except for studies by Cai H. et al. [22], and Guan H. et al. [23]; while in the context of Vietnam, the acquisition cost of original lenvatinib and sorafenib was similar (1,475 USD and 1,583 USD per 28-day cycle, respectively).

This study aimed to evaluate the cost-effectiveness of lenvatinib compared with sorafenib as a first-line treatment in unresectable advanced HCC patients from the perspective of a public third-party payer of Vietnam to inform reimbursement decisions for lenvatinib.

## Methods

### Modelling conceptualization

Systematic reviews on economic evaluations and effectiveness of lenvatinib with searching in e-databases (e.g., PubMed, Cochrane and Scopus) as well as in websites of health technology assessment agencies (i.e., National Institute for Health and Care Excellence – NICE and Canadian Agency for Drugs and Technologies in Health – CADTH) were conducted by the research team to conceptualize the model structure, patients pathway and data sources for efficacy/effectiveness of lenvatinib and health utilities of different health states. A health economic plan was developed based on the literature review and consultations with clinical experts in Vietnam.

### Target population

This study simulated the disease progression of adult patients with unresectable HCC who had not received treatment for advanced disease, which was consistent with the approved indication in Vietnam and the target population in the pivotal clinical trial of lenvatinib (i.e., REFLECT study) [8].

### Intervention and comparator

As previously mentioned, both lenvatinib and sorafenib were recommended as the first-line treatment for advanced stage in HCC patients with ECOG PS 1–2 and Child-Pugh A/B in Vietnam [24], and both medications were authorized in Vietnam. However, only sorafenib was included in the benefit package, with a reimbursement rate of 50%. During a period of more than 10 years since the inclusion of sorafenib in the reimbursement list in Vietnam, no active treatment for advanced HCC patients has been added. Therefore, in this cost-utility analysis, sorafenib was selected as the comparator to reflect the current clinical practice in Vietnam.

Two or three 4-mg lenvatinib tablets were administered for patients based on the patients’ weight as indicated by the manufacturer. Meanwhile, the dosage of sorafenib was 400 mg, twice a day. Patients received lenvatinib or sorafenib until disease progression or intolerance, in line with the REFLECT trial, which reported a longer median treatment duration for lenvatinib than sorafenib (5.7 months vs. 3.7 months).

### Model structure

A three-health state partitioned survival model (PSM) was constructed to simulate the patient pathway. S1 Fig illustrated the model structure, including PFS state, progressed disease (PD) state, and death (S1 Fig).

All patients entered the model in the PFS state, receiving either lenvatinib or sorafenib. Patients in this state could remain in PFS, progress to PD, or die. Patients in PD state could only remain in this state or die but could not move backward to PFS.

The PSM model is commonly used in economic evaluations of cancer drugs, especially in advanced stages where the target of treatment was to delay patients’ progression and prolong survival. This modelling approach was also employed in previous economic evaluations of lenvatinib as the first-line treatment for advanced HCC patients [17,19–21,23] as well as in submissions to health technology assessment agencies such as NICE [25] and CADTH [26].

A time horizon of 10 years with 28-day cycle was applied to estimate costs and health outcomes of two compared groups. Results from the REFLECT trial [8] showed that median OS of patients treated by lenvatinib and sorafenib were 13.6 months and 12.3 months, respectively. At 42 months of follow-up since randomization, only 20% of patients in both groups had survived. Therefore, a time horizon of 10 years, which was similar to previous economic evaluations [20–22], was considered sufficient to capture the long-term costs and benefit of lenvatinib compared to sorafenib.

### Model parameters

Three main groups of input parameters were collected as follows

#### Efficacy and Safety of Lenvatinib and Sorafenib.

Parameters related to the efficacy on PFS and OS, and safety of lenvatinib and sorafenib were obtained from the pivotal REFLECT trial [8]. This study was selected because it was the only head-to-head randomized controlled trial comparing lenvatinib and sorafenib. In addition, this study had a larger sample size and longer follow-up duration as compared to other observational studies in the literature [9–16,27–33].

Parametric survival analyses based on patient-level data from the REFLECT trial were used to extrapolate the efficacy of lenvatinib and sorafenib up to the 10-year time horizon (S2 Fig). Six parametric survival analyses recommended by NICE Decision Support Unit [34] (i.e., Exponential, Weibull, Gompertz, Log-Normal, Log-Logistic and Generalized Gamma) were investigated; the distribution with the lowest Akaike Information Criterion (AIC) was selected for the base-case analysis. Based on the information criteria presented in S1 Table, the Log-logistic distribution was selected to model overall survival (OS) in the base-case analysis. For progression-free survival (PFS), although the Log-normal distribution showed the best fit for lenvatinib and the Gamma distribution for sorafenib, the Log-normal distribution was applied to both arms to ensure model consistency and parsimony in long-term extrapolation. In addition, survival analyses were adjusted for multiple co-variates including baseline AFP level (≥ or < 200 ng/mL), body weight (< 60 or ≥ 60 kg), Child-Pugh score (A, B), ECOG PS (0 or 1), factors of carcinogenesis, involved disease sites (Liver, Lung, Bone, Other), macroscopic portal vein invasion, extrahepatic spread or both (yes, no), region (Asia-Pacific, Western regions). In addition, post-progression treatment was also adjusted for OS, because no active treatment for patients who progressed after the first-line systemic treatment for unresectable HCC was included in the benefit package in Vietnam.

Regarding adverse events (AE) of lenvatinib and sorafenib, only grade 3 or 4 AEs with > 1% difference between the two arms were included in the model (i.e., hand-foot syndrome, hypertension, decreased weight, proteinuria, decreased platelet count, elevated aspartate aminotransferase, and increased blood bilirubin).

### Healthcare resource and cost data

This study was performed from the perspective of the public third-party payer; therefore, only direct medical costs were considered. All costs were converted to USD 2022 (1USD = 23,681 VND) [35].

Acquisition costs of lenvatinib, sorafenib, and other medications were obtained from the bidding results and price negotiations in 2022 [36]. The total drug cost of sorafenib and lenvatinib per cycle was calculated based on the dose intensity observed in REFLECT trial (i.e., 87.7% for patients receiving lenvatinib 8 mg, 87.5% for those receiving lenvatinib 12 mg, and 83.0% for those receiving sorafenib). In addition, for the lenvatinib arm, the proportion of patients with target doses of 8 mg (body weight < 60 kg) was informed by the clinical experts in Vietnam.

From October 2022 to December 2022, healthcare resources for each health state (i.e., PFS and PD), as well as for AEs management were informed by five local clinical experts, who had at least 5-year experience of treatment for advanced HCC patients in five prestigious tertiary or oncology hospitals in Vietnam. Unit costs of each medical service were obtained from the National List of Medical Services issued by the MoH [37].

The study protocol was approved by the ethical committee of Hanoi University of Pharmacy, Hanoi, Vietnam (2209/PCT-HĐĐĐ). Written informed consents were obtained from all clinical experts.

### Utility data

Health state utility values were derived directly from EQ-5D-3L data collected in the pivotal trial REFLECT. Patients completed EQ-5D-3L questionnaires at baseline visit, on day 1 of each subsequent treatment cycle and at the off-treatment visit. Mean utility values and their standard errors for the progression-free and progressed health states were calculated from these data. Utility values were very similar between lenvatinib and sorafenib arms, therefore, the mean utility values in PFS and PD states of overall trial population were used in the model. This approach was applied in previous economic evaluations [17,18,20,23,34].

### Model analyses

The incremental cost-effectiveness ratio (ICER) of lenvatinib compared with sorafenib was calculated and compared to the willingness-to-pay (WTP) threshold 12,160 USD per QALY gained, equivalent to three times Vietnam’s GDP per capita in 2022 (4,053 USD) [38]. The 3 × GDP per capital rule is a widely used proxy where country-specific thresholds are lacking recommended by WHO-CHOICE [39] and also the MoH of Vietnam [40]. Both costs and health outcomes were discounted at a rate of 3% in the base-case analysis as recommended by the MoH. Half-cycle correction was implemented to account for the fact that the transition among different health states could occur at any point during the cycle. The model was built and run using the Microsoft Office Excel 2016.

### Sensitivity analysis

We performed one-way sensitivity analysis to identify the most influential parameters on the ICER; results were summarized in a tornado diagram. The discount rate varied from 0 to 6%, other parameters were varied among their 95% CIs. A standard error of 15% of base-case value was applied if 95% CI was not available.

Probabilistic sensitivity analysis (PSA) with 5,000 simulations was performed to explore the variations of ICER when all parameters varied around their distributions. Results from PSA were illustrated on a cost-effectiveness plane and a cost-effectiveness acceptability curve (CEAC).

In the base-case analysis, the reimbursement rates of investigated drugs were not considered. Therefore, to reflect the current context in which sorafenib was reimbursed at a rate of 50% for advanced HCC patients, we performed scenario analyses with different reimbursement rates of lenvatinib, including 30%, 50% and 70%. These levels were selected to represent commonly observed reimbursement rates for oncology medicines under the Vietnamese public health insurance scheme.

In addition, in the base-case analysis, we applied multivariate adjustment to extrapolate data on efficacy of lenvatinib and sorafenib beyond the follow-up duration in the REFLECT trial. To explore the results in different approaches of extrapolation, we performed scenario analyses without adjustment or only adjustment for AFP-level.

Finally, to explore the impact of the choice of time horizon, we performed scenario analyses in which time horizon varied from 5 to 25 years.

## Results

### Base-case analysis

Values of input parameters as well as their distribution and data sources were summarized in the S2 Table.

**Fig 1:**
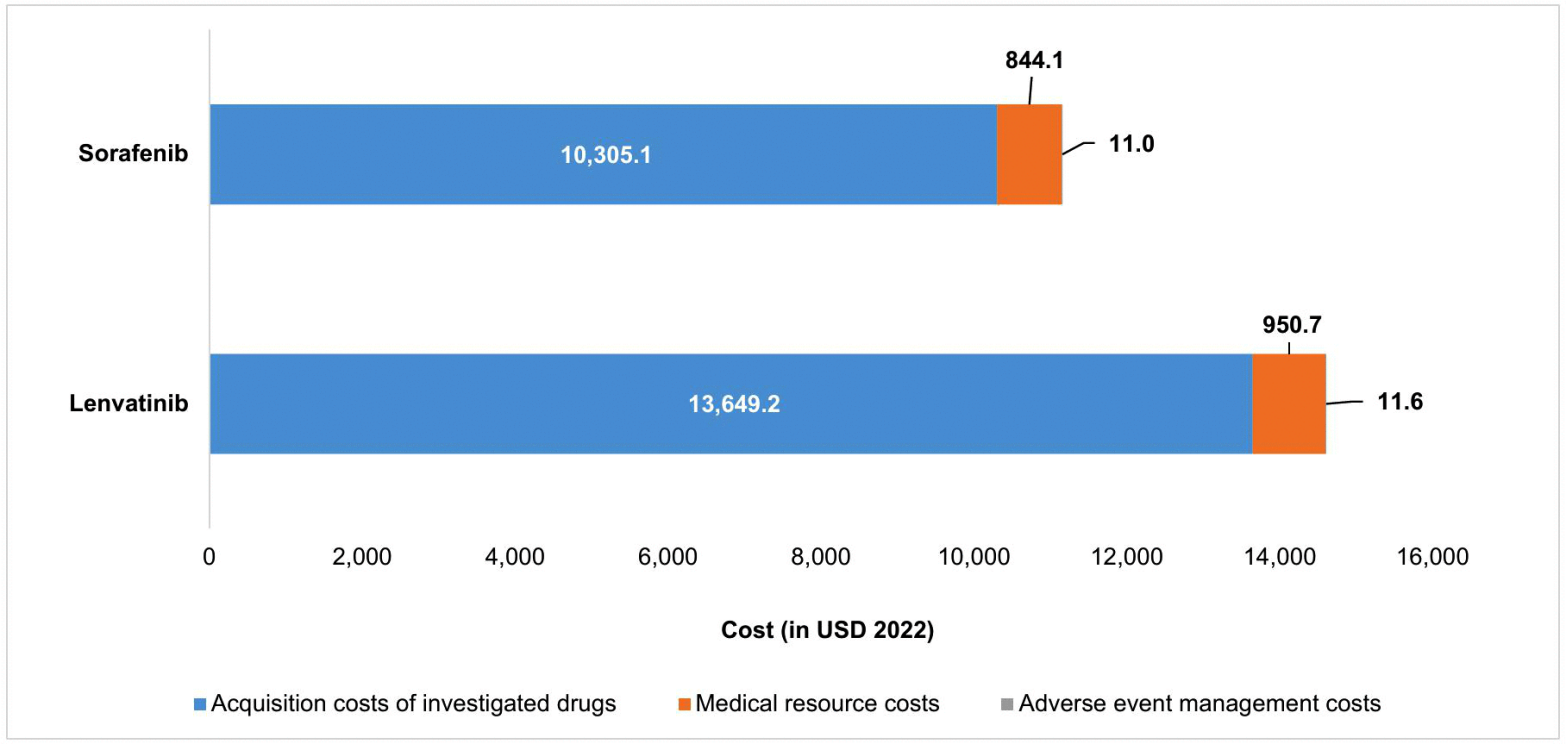
Cost components of two compared arms. This figure illustrates the proportions of acquisition cost of investigated medication, medical resource costs and the adverse event management cost in two compared arms (i.e., lenvatinib and sorafenib).

Discounted average cost per patient receiving lenvatinib was 14,611.6 USD compared with 11,160.2 USD in patients receiving sorafenib (Fig 1). In both arms, acquisition cost of lenvatinib and sorafenib accounted for more than 90% of total costs.

Results from the base-case analysis indicated that the usage of lenvatinib increased total average costs of 3,451.3 USD but gained more 0.28 LYs and 0.21 QALYs as compared with sorafenib. ICER per LY gained was 12,224.7 USD, per QALY gained was 16,114.5 USD (Table 1). The ICER of lenvatinib versus sorafenib was approximately four times the GDP per capita (1 GDP per capita = 4,053 USD), exceeding the recommended WTP of three times GDP per capita (12,160 USD).

**Table 1:** Incremental cost-effectiveness in base-case analysis.

Health outcomes	Lenvatinib	Sorafenib
Progression-free life years	0.88	0.54
Life years	1.62	1.34
QALYs	1.16	0.94
Costs (USD)	14,611.6	11,160.2
Incremental LY	0.28	
Incremental QALYs	0.21	
Incremental Costs	3,451.3	
ICER	12,224.7 USD/LY 16,114.5 USD/QALY	

### Sensitivity analysis

**Fig 2:**
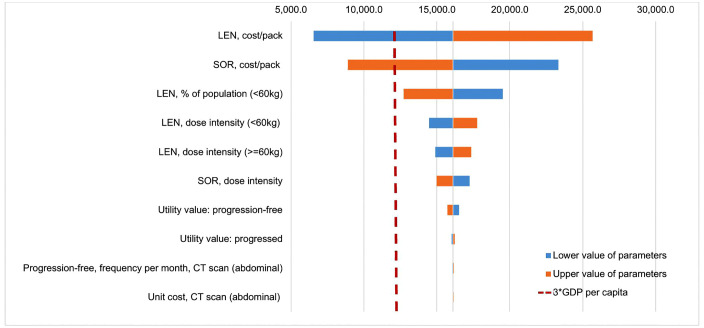
One-way Sensitivity Analysis: Tornado Diagram Showing Most Influential Parameters. This figure illustrates the 10 most influential parameters on the ICER.

Results from one-way sensitivity analysis were illustrated in the Tornado diagram (Fig 2). The variation of acquisition cost of lenvatinib and sorafenib were the two most influential parameters on the ICER per QALY. If the acquisition cost of lenvatinib was reduced by 15% or the acquisition cost of sorafenib increased by 15%, ICERs were less than WTP of 3xGDP per capita. In contrast, the 15% increase of the acquisition cost of lenvatinib or 15% decrease of the acquisition cost of sorafenib led to very high ICER, varying from 23,331.8 USD to 25,673.9 USD, approximately 6xGDP per capita. The variations of other parameters had a less impact on the main outcomes and did not change the conclusion on the cost-effectiveness of lenvatinib versus sorafenib.

**Fig 3:**
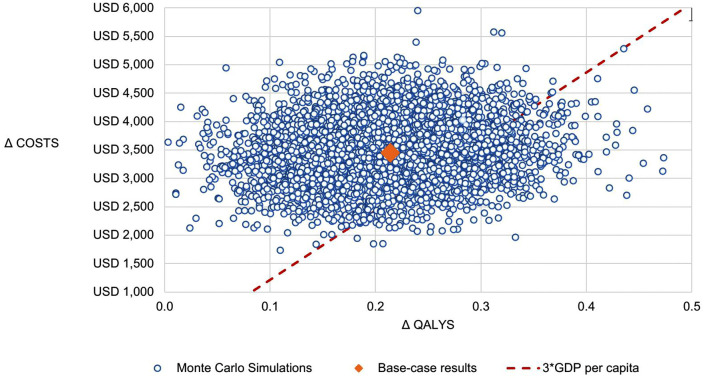
Cost-effectiveness plane. This figure illustrates results from 5,000 simulations in probabilistic analysis.

**Fig 4:**
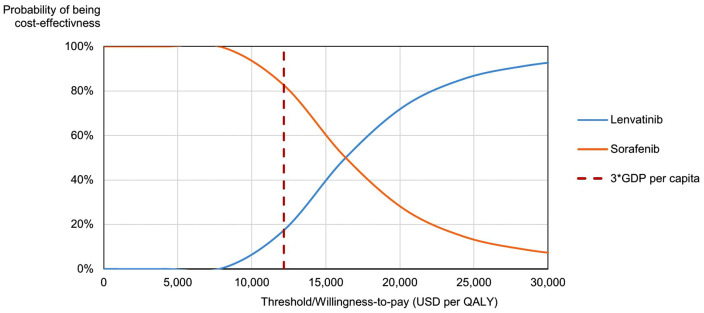
Cost-Effectiveness Acceptability Curve. This figure illustrates the probability of being cost-effective of lenvatinib and sorafenib at different willingness-to-pay threshold.

The average incremental cost of lenvatinib compared with sorafenib from 5,000 simulations was 3,475.1 USD with an increased QALYs of 0.21 equaling to an ICER of 16,270.3 USD per QALY, which was consistent with the base-case analysis. The results of the probabilistic sensitivity analysis were depicted using a cost-efectiveness plane (Fig 3) and a cost-efectiveness acceptability curve (Fig 4). In the cost-efectiveness plane, most simulations fell in the north-east quadrant, indicating that lenvatinib was more effective and more costly than sorafenib. For simulations where the ICER was below the specified willingness-to-pay (WTP) threshold, lenvatinib was considered the preferred option. At a WTP threshold of three times GDP per capita (12,160 USD), lenvatinib had a 17.4% probability of being cost-effective compared to sorafenib (Fig 3). The acceptability curve (Fig 4) demonstrated that this probability increased with higher WTP thresholds, rising to 49.1% at four times GDP per capita (16,214 USD) and exceeding 98% at eleven times GDP per capita (44,407 USD).

To reflect the current context in which sorafenib was reimbursed at a rate of 50% for patients with advanced HCC, we conducted scenario analyses assuming different reimbursement rates for lenvatinib, including 30%, 50% and 70%. Table 2 presented the results of these scenarios, illustrating the impact of varying reimbursement rates for lenvatinib on its cost-effectiveness. If the reimbursement rate of lenvatinib was higher than that of sorafenib (i.e., 70% vs 50%, respectively), the ICER increased to 21,053.5 USD per QALY (~5.2xGDP per capita) or lenvatinib was not cost-effective compared with sorafenib. However, with the same reimbursement rate of 50%, ICER of lenvatinib to sorafenib was 8,307.6 USD per QALY, which was less than the recommended WTP. If the reimbursement rate of lenvatinib was only 30%, lenvatinib gained more QALYs with lower costs, or was dominant to sorafenib.

**Table 2:** Incremental cost-effectiveness in scenario analyses.

Reimbursement rate of lenvatinib	Inc. Costs	ICER		Conclusion
**VND**	**GDP per capita**
70%	4,509.1	21,053.5	5.2	Not cost-effectiveness
50%	1,779.3	8,307.6	2.1	Cost-effectiveness
30%	−950.5	Dominant	Dominant	Dominant

*The price of sorafenib was set at 50% of the base-case value in this scenario analyses*

*Inc.: Incremental; VND: Vietnam Dong; GDP: Gross Domestic Product; ICER: Incremental cost-effectiveness ratio*

In addition, in the base-case analysis, we applied multivariate adjustment to extrapolate the survival outcomes of lenvatinib and sorafenib beyond the trial period. To assess the robustness of our findings, we also conducted scenario analyses using alternative extrapolation approaches, either no adjustment or adjustment only for AFP level. As shown in S3 Table, these assumptions resulted in only minor variations in the ICERs (ranging from 14,637.1 to 15,591.8 USD per QALY). We also explored the impact of varying the time horizon from 5 to 25 years. Shortening the time horizon to 5 years increased the ICER to 19,051.2 USD/QALY, while extending it led to lower ICERs, with the value decreasing to 14,876.9 USD/QALY at a 25-year horizon. Despite these changes, all ICERs remained above the WTP threshold, and the conclusion that lenvatinib is not cost-effective compared with sorafenib was consistent across scenarios and aligned with the base-case analysis (S3 Table).

## Discussion

This study evaluated the cost-effectiveness of lenvatinib compared to sorafenib in unresectable HCC patients as a first-line treatment from perspective of Vietnam – a LMIC with a high burden of HCC. Results from this study indicated that, without consideration of the reimbursement rate of the two compared drugs, lenvatinib was not cost-effective compared with sorafenib, with an ICER of 16,114.5 USD per QALY. However, with the same reimbursement rate as sorafenib of 50%, lenvatinib was cost-effective with an ICER of 8,307.6 USD per QALY.

The result from base-case analysis of our study indicated that the use of lenvatinib gained 0.21 QALYs compared with sorafenib, which was similar to findings from study by Kobayashi M. et al. [19], slightly lower than that in study by Ikeda S. et al. [17], but was much lower than that reported in study by Cai et al. [22]. Ikeda S. et al. [17] performed evaluation based on PSM using the efficacy data from Japanese patients in the REFLECT trial. In this Japanese subgroup, the efficacy of lenvatinib over sorafenib on PFS and OS tended to be better than in the overall population, which probably led to higher LYs and QALYs gained [8,17]. In the study by Cai et al. [22], the authors applied Markov model and use Weibull distributions to extrapolate the data on efficacy beyond the follow-up period. In addition, the utility values in PFS and PD states used in study by Cai et al. [22] were also higher than those in our study. Those differences might lead to higher QALY reported in study by Cai et al.

Notably, the conclusion on the cost-effectiveness of lenvatinib over sorafenib in the base-case analysis in our study was different from previous economic evaluations [17–23], which indicated that lenvatinib was dominant or cost-effective compared with sorafenib. It should be noted that the acquisition cost of lenvatinib in previous economic evaluations [17–21,23] was much lower than that of sorafenib; in contrast, in our study there was no significant difference among two compared drugs (i.e., 1,474.6 USD vs 1,583.3 USD per 28-day cycle). As indicated in the one-way sensitivity analysis, the acquisition costs of lenvatinib and sorafenib were the most influential parameters on ICER. Therefore, the main reason for the contradictory conclusions of the cost-effectiveness of lenvatinib from our study and previous economic evaluations might be due to the difference in terms of medication costs in different settings.

### Limitations

Our study modelled the progression of unresectable HCC patients based on the model structures from previous studies and opinions of clinical experts in Vietnam to capture the main health states and important clinical outcomes of this targeted population. However, some limitations of this study should be discussed.

Due to the lack of real-world data on the efficacy, safety and health-related quality of life (i.e., utility values for QALY estimation) of lenvatinib in the Vietnamese population, we borrowed data from the pivotal clinical trial REFLECT [8]. Data from this multicenter trial might not perfectly reflect the effectiveness of lenvatinib and sorafenib in Vietnamese population, which may limit the generalizability of the cost-effectiveness results to the local context. However, it should be noted that the subgroup analysis from the REFLECT trial indicated that effectiveness of lenvatinib over sorafenib seemed to be better in the Asia-Pacific population.

In addition, since no active drug treatment for patients who progressed after first-line systemic therapy for unresectable HCC is included in the benefit package in Vietnam and given that our analysis was conducted from a third-party payer perspective, we excluded drug costs for post-progression treatment. This may limit the generalizability of our findings to private sector patients who are able and willing to pay for such treatments out-of-pocket.

One limitation of the probabilistic sensitivity analysis was that utility values for the progression-free and progressed disease states were sampled independently. As a result, due to overlapping 95% confidence intervals, there may be situations where the utility for the progressed disease state exceeds that of the progression-free state. However, the probability of such overlap is expected to be quite low and therefore not a major concern.

All costs and medical resources used in each health state and for managing AE were estimated based on clinical experts’ opinions; therefore, the data may not be representative of the whole population. To deal with uncertainty of input parameters and model structure, various sensitivity analyses were conducted. The results from those analyses proved the consolidation of base-case findings.

### Implementation recommendations

Results from our study indicated that, from the perspective of the third-party payer, at a similar reimbursement rate of sorafenib, lenvatinib was cost-effective. In the local setting, only sorafenib was included in the benefit package. It should be noted that, during a period of more than 10 years since the approval of sorafenib, no medication except lenvatinib had proved to be non-inferior or superior to sorafenib in terms of OS, or to significantly prolonged PFS [8,41–43]. Lenvatinib has been authorized for the Vietnamese market since 2020, however, this medication has not yet been included in the benefit package. The findings from this study suggested that lenvatinib should be considered for reimbursement to provide additional options for both patients and physicians in the local context. However, to ensure the sustainability of the public insurance fund, evidence from a budget impact analysis should be considered to make decision on the reimbursement rate of lenvatinib.

## Conclusion

In the context of Vietnam, lenvatinib was cost-effective compared with sorafenib at the WTP of 3xGDP per capita if this medication was reimbursed at the rate of 50%, which was similar to the current reimbursement rate of sorafenib. To inform the reimbursement rate for lenvatinib, in addition to the evidence from this economic evaluation, the impact of adding lenvatinib in the benefit package on the public insurance fund should be considered to provide additional options for clinical experts and patients while ensuring sustainability of public health system.

## Supporting information

S1 Fig Model structure. This figure illustrated patient pathway with 3 health states, including progression-free survival, progression disease and death.
(DOCX)

S2 Fig Extrapolation data on PFS and OS. (DOCX)

S1 Table Goodness-of-fit statistics for parametric survival models of OS and PFS. (DOCX)

S2 Table Input parameters: Values and ranges. (DOCX)

S3 Table Results from scenario analysis. (DOCX)

S1 Checklist CHEERS Checklist_2022. (DOCX)
