# Supplementary figures and images for: Cost-utility analysis of lenvatinib and sorafenib for the first-line treatment of unresectable hepatocellular carcinoma in Vietnam: Evidence from a lower-middle income country
Source: PLoS One. 2026 Apr 3;21(4):e0345212. doi: 10.1371/journal.pone.0345212 (PMC13048410; doi:10.1371/journal.pone.0345212)

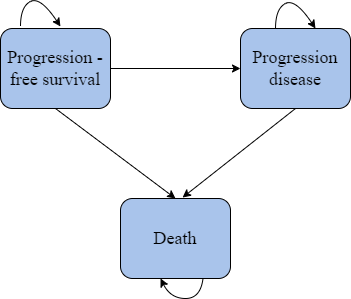


**S1 Fig. Model structure**

Supplement: S1 Fig — This figure illustrated patient pathway with 3 health states, including progression-free survival, progression disease and death. (DOCX) [file pone.0345212.s001.docx]

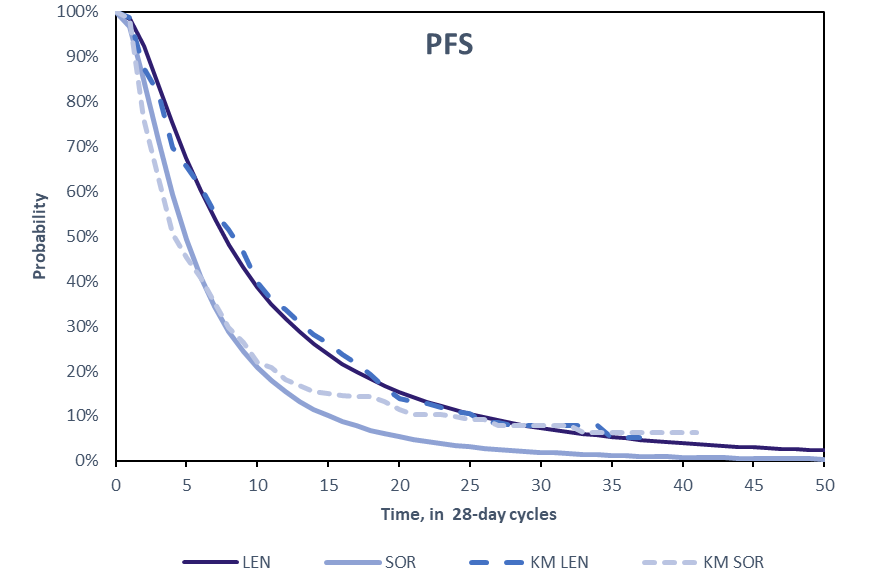


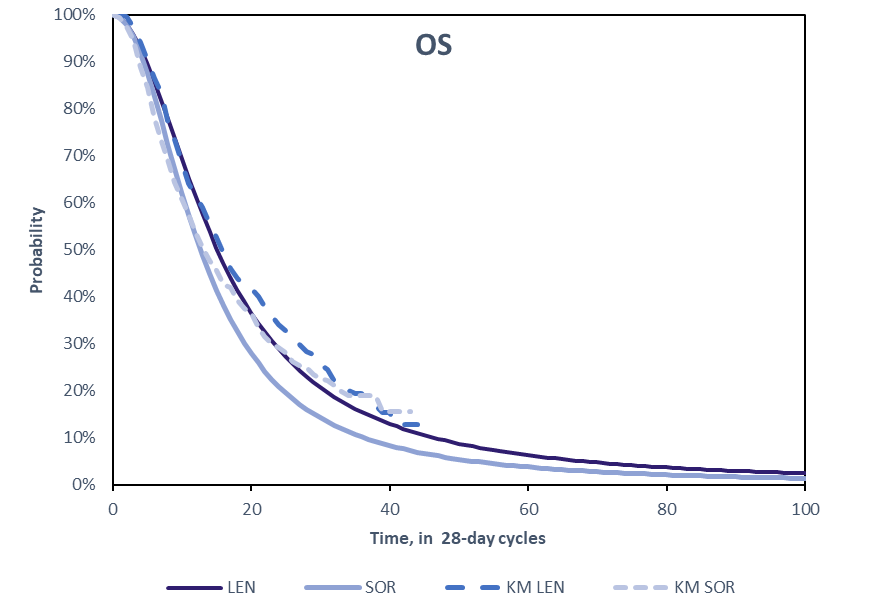


**S2 Fig. Extrapolation data on PFS and OS**

Supplement: S2 Fig — (DOCX) [file pone.0345212.s002.docx]
