# Supplementary material for: Cost-utility analysis of lenvatinib and sorafenib for the first-line treatment of unresectable hepatocellular carcinoma in Vietnam: Evidence from a lower-middle income country
Source: PLoS One. 2026 Apr 3;21(4):e0345212. doi: 10.1371/journal.pone.0345212 (PMC13048410; doi:10.1371/journal.pone.0345212)
Supplement: S1 Table — (DOCX) [file pone.0345212.s003.docx]

**S1 Table. Goodness-of-fit statistics (AIC and BIC) for parametric survival models of OS and PFS**

| Overall survival (OS) | Lenvatinib | | Sorafenib | |
| --- | --- | --- | --- | --- |
|  | **AIC** | **BIC** | **AIC** | **BIC** |
| Exponential | 1216.614 | 1266.624 | 1200.526 | 1250.436 |
| Weibull | 1175.486 | 1229.663 | 1154.979 | 1209.047 |
| Gamma | 1160.386 | 1218.731 | 1132.919 | 1191.147 |
| Gompertz | 1200.253 | 1254.431 | 1188.415 | 1242.483 |
| Log-normal | 1162.903 | 1217.081 | 1133.225 | 1187.294 |
| Log-logistic | **1158.832** | **1213.010** | **1128.983** | **1183.051** |
| Progression-free survival (PFS) | **Lenvatinib** | | **Sorafenib** | |
|  | **AIC** | **BIC** | **AIC** | **BIC** |
| Exponential | 1168.173 | 1218.184 | 1208.589 | 1258.498 |
| Weibull | 1143.463 | 1197.641 | 1195.242 | 1249.310 |
| Gamma | **1116.269 (2)** | **1174.615 (2)** | **1062.170 (1)** | **1120.397 (1)** |
| Gompertz | 1163.856 | 1218.033 | 1206.130 | 1260.198 |
| Log-normal | **1114.546 (1)** | **1168.724 (1)** | **1098.051 (2)** | **1152.119 (2)** |
| Log-logistic | 1121.265 | 1175.442 | 1102.300 | 1156.368 |

*AIC: Akaike Information Criterion; BIC: Bayesian Information Criterion.*
