# Supplementary material for: Cost-utility analysis of lenvatinib and sorafenib for the first-line treatment of unresectable hepatocellular carcinoma in Vietnam: Evidence from a lower-middle income country
Source: PLoS One. 2026 Apr 3;21(4):e0345212. doi: 10.1371/journal.pone.0345212 (PMC13048410; doi:10.1371/journal.pone.0345212)
Supplement: S2 Table — (DOCX) [file pone.0345212.s004.docx]

**S2 Table. Input parameters: Values and ranges**

| **Input Parameters** | **Value** | **Lower value** | **Upper value** | **Standard error** | **Distribution** | **Source** |
| --- | --- | --- | --- | --- | --- | --- |
| **Overall survival (Log-logistic)** | | | | | | |
| **Lenvatinib** | | | | | | |
| *Coefficient* | | | | | | |
| -Region | 0.09 |  |  | 0.10 | Multivariate normal | Individual patient data from REFLECT trial |
| -Macroscopic portal vein invasion or extrahepatic spread | 0.3 |  |  | 0.11 |  |  |
| -ECOG performance status | -0.18 |  |  | 0.09 |  |  |
| -Body weight | 0.06 |  |  | 0.09 |  |  |
| -Alpha-fetoprotein | -0.36 |  |  | 0.09 |  |  |
| -Child-Pugh score | -2.43 |  |  | 0.56 |  |  |
| -Etiology of HCC | 0.01 |  |  | 0.09 |  |  |
| -Involved disease in the liver | -0.74 |  |  | 0.18 |  |  |
| -Involved disease in the lung | -0.03 |  |  | 0.11 |  |  |
| -Involved disease in the bone | -0.29 |  |  | 0.14 |  |  |
| -Involved disease in other sites | -0.18 |  |  | 0.12 |  |  |
| -Post-progression therapy | 0.47 |  |  | 0.09 |  |  |
| *Constant* | 3.21 |  |  | 0.22 |  |  |
| *ln(gamma) coefficient* | -0.67 |  |  | 0.04 |  |  |
| **Sorafenib** | | | | | | |
| *Coefficient* |  |  |  |  |  |  |
| -Region | 0.22 |  |  | 0.1 | Multivariate normal | Individual patient data from REFLECT trial |
| -Macroscopic portal vein invasion or extrahepatic spread | 0.29 |  |  | 0.09 |  |  |
| -ECOG performance status | -0.04 |  |  | 0.09 |  |  |
| -Body weight | -0.02 |  |  | 0.09 |  |  |
| -Alpha-fetoprotein | -0.48 |  |  | 0.08 |  |  |
| -Child-Pugh score | -1.00 |  |  | 0.35 |  |  |
| -Etiology of HCC | -0.1 |  |  | 0.09 |  |  |
| -Involved disease in the liver | -0.70 |  |  | 0.16 |  |  |
| -Involved disease in the lung | -0.21 |  |  | 0.09 |  |  |
| -Involved disease in the bone | -0.39 |  |  | 0.14 |  |  |
| -Involved disease in other sites | -0.13 |  |  | 0.10 |  |  |
| -Post-progression therapy | 0.54 |  |  | 0.08 |  |  |
| *Constant* | 3.09 |  |  | 0.19 |  |  |
| *ln(gamma) coefficient* | -0.74 |  |  | 0.04 |  |  |
| **Progression-free survival (Log-normal)** | | | | | | |
| **Lenvatinib** |  |  |  |  |  |  |
| *Coefficient* |  |  |  |  |  |  |
| -Region | 0.01 |  |  | 0.11 | Multivariate normal | Individual patient data from REFLECT trial |
| -Macroscopic portal vein invasion or extrahepatic spread | 0.24 |  |  | 0.10 |  |  |
| -ECOG performance status | -0.16 |  |  | 0.09 |  |  |
| -Body weight | 0.10 |  |  | 0.10 |  |  |
| -Alpha-fetoprotein | -0.34 |  |  | 0.09 |  |  |
| -Child-Pugh score | -1.80 |  |  | 0.49 |  |  |
| -Etiology of HCC | -0.01 |  |  | 0.10 |  |  |
| -Involved disease in the liver | -0.13 |  |  | 0.15 |  |  |
| -Involved disease in the lung | -0.05 |  |  | 0.10 |  |  |
| -Involved disease in the bone | -0.27 |  |  | 0.15 |  |  |
| -Involved disease in other sites | 0.10 |  |  | 0.11 |  |  |
| *Constant* | 2.16 |  |  | 0.18 |  |  |
| *ln(gamma) coefficient* | -0.06 |  |  | 0.04 |  |  |
| **Sorafenib** | | | | | | |
| *Coefficient* |  |  |  |  |  |  |
| -Region | 0.29 |  |  | 0.11 | Multivariate normal | Individual patient data from REFLECT trial |
| -Macroscopic portal vein invasion or extrahepatic spread | 0.24 |  |  | 0.10 |  |  |
| -ECOG performance status | 0.03 |  |  | 0.09 |  |  |
| -Body weight | -0.06 |  |  | 0.10 |  |  |
| -Alpha-fetoprotein | -0.39 |  |  | 0.09 |  |  |
| -Child-Pugh score | -0.48 |  |  | 0.49 |  |  |
| -Etiology of HCC | -0.07 |  |  | 0.10 |  |  |
| -Involved disease in the liver | -0.61 |  |  | 0.15 |  |  |
| -Involved disease in the lung | -0.29 |  |  | 0.10 |  |  |
| -Involved disease in the bone | -0.33 |  |  | 0.15 |  |  |
| -Involved disease in other sites | -0.17 |  |  | 0.11 |  |  |
| *Constant* | 2.27 |  |  | 0.18 |  |  |
| *ln(sigma) coefficient* | -0.14 |  |  | 0.04 |  |  |
| **Incidence of grade ≥ 3 adverse events (%)** | | | | | | |
| **Lenvatinib** | | | | | | |
| Palmar-plantar erythrodysaesthesia | 2.9 | 1.6 | 4.9 |  | Beta | REFELCT trial ^1^ |
| Hypertension | 23.3 | 19.6 | 27.4 |  | Beta |  |
| Weight decreased | 7.6 | 5.4 | 10.3 |  | Beta |  |
| Proteinuria | 5.7 | 3.8 | 8.1 |  | Beta |  |
| Decreased platelet count | 5.4 | 3.6 | 7.9 |  | Beta |  |
| Elevated aspartate aminotransferase | 5.0 | 3.3 | 7.4 |  | Beta |  |
| Increased blood bilirubin | 6.5 | 4.5 | 9.1 |  | Beta |  |
| **Sorafenib** | | | | | | |
| Palmar-plantar erythrodysaesthesia | 11.4 | 8.7 | 14.6 |  | Beta | REFELCT trial ^1^ |
| Hypertension | 14.3 | 11.3 | 17.8 |  | Beta |  |
| Weight decreased | 2.9 | 1.6 | 4.9 |  | Beta |  |
| Proteinuria | 1.7 | 0.7 | 3.3 |  | Beta |  |
| Decreased platelet count | 3.4 | 1.9 | 5.4 |  | Beta |  |
| Elevated aspartate aminotransferase | 8.0 | 5.7 | 10.8 |  | Beta |  |
| Increased blood bilirubin | 4.8 | 3.1 | 7.2 |  | Beta |  |
| **Drug acquisition cost (USD)** | | | | | | |
| Lenvatinib (20 capsules per pack) | 544 |  | | ± 15% | Not applicable | Bidding cost |
| Sorafenib (60 tablets per pack) | 1,022 |  |  |  |  |  |
| **Dose intensity** | | | | | | |
| Lenvatinib (target dose 8 mg) | 0.88 |  | | 0.02 | Beta | Individual patient data from REFLECT trial |
| Lenvatinib (target dose 12 mg) | 0.88 |  |  | 0.03 |  |  |
| Sorafenib | 0.83 |  |  | 0.01 |  |  |
| **Health state costs (USD)** | | | | | | |
| Progression-free disease state | 33,6 |  | | ± 15% | Gamma |  |
| Progressed-disease state | 56,6 |  |  |  | Gamma |  |
| **Adverse events costs (USD)** | | | | | | |
| Palmar-plantar erythrodysaesthesia | 17,0 |  | | ±50% | Gamma | Experts consulations |
| Hypertension | 13,3 |  |  |  | Gamma |  |
| Increased blood bilirubin | 51,0 |  |  |  | Gamma |  |
| Decreased platelet count | 17,5 |  |  |  | Gamma |  |
| Elevated aspartate aminotransferase | 26,9 |  |  |  | Gamma |  |
| **Health state utilities** | | | | | | |
| Progression-free disease state | 0.75 |  |  | 0.0079 | Beta | Individual patient data from REFLECT trial |
| Progressed-disease state | 0.68 |  |  | 0.0118 | Beta |  |
| *Notes: ECOG - Eastern Cooperative Oncology Group; HCC - hepatocellular carcinoma; VND: Vietnamese currency* | | | | | | |

**Reference**

1. Kudo M, Finn RS, Qin S, et al. Lenvatinib versus sorafenib in first-line treatment of patients with unresectable hepatocellular carcinoma: a randomised phase 3 non-inferiority trial. The Lancet 2018;391(10126):1163-73.
