# Supplementary material for: Cost-utility analysis of lenvatinib and sorafenib for the first-line treatment of unresectable hepatocellular carcinoma in Vietnam: Evidence from a lower-middle income country
Source: PLoS One. 2026 Apr 3;21(4):e0345212. doi: 10.1371/journal.pone.0345212 (PMC13048410; doi:10.1371/journal.pone.0345212)
Supplement: S3 Table — (DOCX) [file pone.0345212.s005.docx]

**S3 Table. Results from scenario analyses**

| **Scenarios** | **Inc. QALYs** | **Inc. Costs** | **ICER (USD/QALY)** |
| --- | --- | --- | --- |
| Base-case results  (Multivariate adjustment, Time horizon of 10 years) | 0.21 | 3,451.3 | 16,114.5 |
| **Different approach to extrapolate survival data** | | | |
| No adjustment | 0.19 | 2,886.4 | 15,591.8 |
| Only adjustment for AFP level | 0.22 | 3,224.2 | 14,637.1 |
| **Time horizon** | | | |
| Time horizon of 5 years | 0.18 | 3,419.5 | 19,051.2 |
| Time horizon of 15 years | 0.23 | 3,462.7 | 15,362.9 |
| Time horizon of 20 years | 0.23 | 3,468.1 | 15,044.4 |
| Time horizon of 25 years | 0.23 | 3,471.1 | 14,876.9 |

*Inc.: Incremental; QALY: Quality-adjusted life years; ICER: Incremental Cost-Effectiveness Ratio.*
